# *Drosophila* relics *hobo* and *hobo*-MITEs transposons as raw material for new regulatory networks

**DOI:** 10.1590/1678-4685-GMB-2017-0068

**Published:** 2018-03-26

**Authors:** Elgion L.S. Loreto, Maríndia Deprá, José F. Diesel, Yanina Panzera, Vera Lucia S. Valente-Gaiesky

**Affiliations:** 1 Programa de Pós-Graduação em Genética e Biologia Molecular, Universidade Federal do Rio Grande do Sul (UFRGS), Porto Alegre, RS, Brazil; 2 Departamento de Bioquímica e Biologia Molecular (CCNE), Universidade Federal de Santa Maria (UFSM), Santa Maria, RS, Brazil; 3 Departamento de Genética, Universidade Federal do Rio Grande do Sul (UFRGS), Porto Alegre, RS, Brazil; 4 Departamento de Genetica, Universidad de la República de Uruguay (UDELAR), Montevideo, Uruguay

**Keywords:** hobo, transposable elements, *cis*-regulatory sequences, Drosophila

## Abstract

Hypermutable strains of *Drosophila simulans* have been studied for 20 years. Several mutants were isolated and characterized, some of which had phenotypes associated with alteration in development; for example, showing ectopic legs with eyes being expressed in place of antennae. The causal agent of this hypermutability is a non-autonomous *hobo*-related sequence (*hobo*VA). Around 100 mobilizable copies of this element are present in the *D. simulans* genome, and these are likely mobilized by the autonomous and canonical *hobo* element. We have shown that *hobo*VA has transcription factor binding sites for the developmental genes, *hunchback* and *even-skipped,* and that this transposon is expressed in embryos, following the patterns of these genes. We suggest that *hobo* and *hobo*-related elements can be material for the emergence of new regulatory networks.

## Introduction

The eukaryotic genomes sequenced thus far have shown that substantial portions of them are formed by transposable elements (TEs). These elements are extremely variable, and usually, the genomes are composed by dozens of different TE families, which are often represented by degenerated and inactive copies (review in Wicker *et al.*, 2007). TEs have parasitic characteristics, harboring mechanisms that enable them to self-multiply faster than the “host genome”. Furthermore, TEs are an important source of genetic variability to drive evolution. There are many ways that TEs can generate variability; for example, promoting mutations in coding or regulatory regions of genes, chromosome rearrangements, epigenetic alterations and others (reviews in Biémont and Vieira, 2006; Hua-Van *et al.*, 2011).

Recently, a growing body of evidence indicates that TEs are involved in rewiring gene regulatory networks (Feschotte, 2008). TEs typically carry a collection of regulatory elements, such as promotors, *cis*-regulatory sequences, enhancers, insulators, splice and poly(A) sites, usually used for their own expression. Also important in gene regulation involving TEs are those using miRNAs in the pre-translation process or in heterochromatin formation (Feschotte and Gilbert, 2012; Rebollo *et al.*, 2012; Chuong *et al.*, 2017).

This review will focus on *cis*-regulatory sequences, in particular on the potential of *hobo* relics elements to provide sequences for producing mutations in developmentally regulated genes or sequences in which developmental genes can act.

TEs can harbor many transcription factor binding sites (TFBSs) and, the mobile nature of TEs, which allows them to occupy almost any site of a genome, makes them a powerful route for the spread of “ready-to-use” *cis*-regulatory sequences. The addition of new TFBSs in regulatory regions can create novel patterns of gene expression. There are examples in diverse organisms of genes that have exapted TE-TFBSs (review in Chuong *et al.*, 2017). In mammals, Polavarapu *et al.* (2008) found that 7-10% of experimentally characterized TFBSs in the human genome are derived from TEs. Sundaram *et al.* (2014) studied 26 pairs of orthologous transcription factors (TFs) in two pairs of human and mouse cell lines and showed that 20% of binding sites were embedded within TEs. The expression of the human tumor suppressor protein, p53, is regulated by the p53 TFBS found in LTR (long terminal repeats) of *ERV* elements (Wang *et al.*, 2007). In insects, the domestication of the silkworm (*Bombyx mori*) involved the insertion of a partial TE (Taguchi) in *cis*-regulatory region of the ecdysone oxidase (EO) gene, enhancing the expression of this gene. It promotes a developmental uniformity of silkworm individuals, which is a desirable trait for domestication (Sun *et al.*, 2014). The addition of new *cis*-elements from TEs on Cyps genes has been associated with the upregulation of these genes and consequent development of insecticide resistance. Different TEs or TEs regulatory sequences have been linked to this phenomenon as, for example, the retrotransposons *Accord* and *HMS-Beagle*, the transposons *P* and *BARI*, and the helitron *DNAREP1* (Chung *et al.*, 2007; Schmidt *et al.*, 2010; Carareto *et al.*, 2014). In plants, many published examples describe the exaptation of TEs *cis-*regulatory regions. For instance, as the C4 photosynthesis system evolved, many genes involved in it acquired regulatory *cis*-elements from TEs (Cao *et al.*, 2016); and the *hAT* element *Moshan*, from *Prunus*, has *cis*-acting elements, recognized by MYB and WRKY transcription factors (TFs) (Wang *et al.*, 2016). Some transcription factors are products of the so-called “master regulatory genes”, originally defined by Susumu Ohno as “genes that occupy the very top of a regulatory hierarchy” acting over multiple downstream genes directly or through a cascade of gene expression changes (Ohno, 1979). Transposable elements that have TFBSs sensible to master genes are promising for producing evolutionary novelty. As stated by Britten and Davidson (1971), “major events in evolution require significant changes in patterns of gene regulation. These changes most likely consist of additions of novel patterns of regulation or reorganization or pre-existing patterns”. The *hobo* element of *Drosophila* has TFBSs for some master developmental genes and is potentially able to produce remarkable mutations. This can be an interesting example, as some evolutionary novelty can arise.

## Hobo, its relics and MITEs

The *hobo* transposon is a class II transposable element and a member of the *hAT* superfamily (Wicker *et al.*, 2007). The main characteristics of this superfamily are: i) presence of short terminal inverted repeats (TIRs), 10-25 bp in length; ii) target site duplications (TSDs) of 8 bp as a consequence of the transposition process; iii) when complete, elements encode for a transposase of 500-800 amino acids; and iv) different elements of this superfamily share between 20 and 60% of amino acid transposase sequence similarity. This enzyme has an amino acid triad (DDE or DDD) in its catalytic domain (Ladevèze *et al.*, 2012). The *hAT* superfamily is also characterized as being widely present in eukaryotes (Calvi *et al.*, 1991).

Currently, it is proposed that the *hAT* superfamily is formed by three families; *Ac, Buster* and *Tip* (Rossato *et al.*, 2014). In *Drosophila*, the *Ac* family is more representative: in 12 analyzed *Drosophila* genomes, members of this family were found in 11, corresponding to 39 different *hAT* elements, of which 29 were potentially autonomous. However, as the elements are found as multiple copies, most (92.9%) are non-autonomous (Ortiz and Loreto, 2009). The *Buster* family is represented in the *Drosophila* genus only by the *Mar* element, present in species of the *willistoni* group, mainly as MITEs (Deprá *et al.*, 2012). The only element of the *Tip* family described in *Drosophila* so far is *But2,* occurring in some species of groups *melanogaster*, *repleta*, and *willistoni* (Rossato *et al.*, 2014).

A remarkable characteristic of many *hAT* elements is the formation of short, but mobilizable elements, the “Miniature Inverted repeat TEs” (MITEs). They normally have less than 800 bp, with no coding capacity, but with conserved TIRs, and often reach high copy numbers in the genomes (Feschotte and Pritham 2007). In *Drosophila*, 68% of the described elements of the *Ac* family have potentially mobilizable elements with less than 600 bp (Ortiz *et al.*, 2010). Also, the MITEs copies are the most abundant in *Buster* and *Tip* family (Deprá *et al.*, 2012; Rossato *et al.*, 2014).

**Figure 1:**
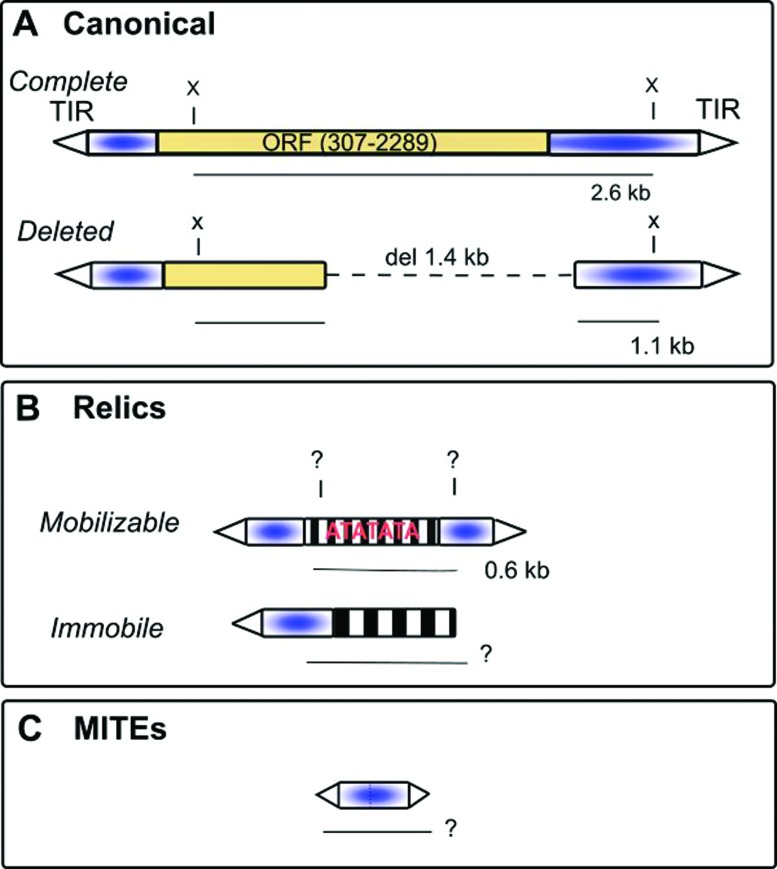
Hobo, relics and MITEs. A) Two forms of canonical *hobo*; the complete and deleted elements. Open triangle = TIR (terminal inverted repeats); *complete* elements have a transposase gene (ORF); *deleted* elements normally lack the central part of the sequence (del 1.4 kbp); X= *Xho*I restriction site, which produces a 2.6 kbp fragment in complete elements and, generally, a fragment of 1.1 kbp in deleted elements. These fragments are used to identify complete and deleted elements in Southern Blots studies; B) Relics *hobo* elements are present in two forms: *mobilizable*, those that have TIRs and conserved subterminal sequences; and *immobile* elements are defective in one TIR. The inner parts of elements are degenerated (striped) and can be AT rich. The *Xho*I site may or may not be present (indicated by “?”). In *D. simulans,* when the *Xho*I site is present, the more abundant relic element generates a 0.6 kbp fragment. The lengths of fragments generated by immobile copies are variable (?) C) *hobo* elements can be found as **MITEs** (80-700 bp).

The *hobo* element belongs to *Ac* family and was discovered in *D. melanogaster* by McGinnis *et al.* (1983), as a 1.3 kbp sequence inserted in the *Sgs-4* gene. Soon after, a complete and active element was described and shown as able to produce hybrid dysgenesis (Blackman *et al.*, 1987), and was used as a vector for genetic transformation (Blackman *et al.*, 1989). This 2,959 bp active *hobo*, called a canonical element, presents an ORF encoding a TPase, short TIRs of 12 bp, and produces a target site duplication (TSD) of 8 bp (Figure 1A). Complete canonical elements have two sites for the restriction enzyme *Xho*I, producing a 2.6 kbp diagnostic band in Southern blot analyses. Population studies showed that some populations had a 2.6-kbp band of complete elements, called H *(hobo)*, and other populations had no band, called E (empty). Short bands resulting from internally deleted elements can be present; the most frequent being elements that produce a 1.1 kbp band in Southern blot analyses (Daniels *et al.*, 1990; Periquet *et al.*, 1990, 1994) (Figure 1A). A second form of the *hobo* element is called “relics” (Figure 1B). Even E populations show, in Southern blots, bands with high molecular size, which had lost *Xho*I sites and were characterized as degenerate sequences, diverging in 10-20% of the canonical elements (Simmons, 1992). A third form is the miniature inverted-repeat transposable element, MITE (Figure 1C) (Ortiz and Loreto, 2008). MITEs are characteristically 80-500 bp in size (but they can sometimes reach lengths of up to 1.6 kbp).

The canonical *hobo* is also found in *D. simulans* and *D. mauritiana* (Boussy and Daniels, 1991). The high similarity observed between the sequences of this element in these species led Simmons (1992) to suggest that horizontal transfer could have occurred for this TE between these species. The “relics” *hobo* has a wide distribution. Although it is mainly restricted to the *melanogaster* subgroup, these sequences are present in *D. melanogaster*, *D. simulans*, *D. sechellia*, *D. mauritiana*, *D. santomea*, *D. yakuba*, *D. teissieri* and *D. erecta* (Ortiz and Loreto, 2008).

## A hypermutable strain and the occurrence of developmental mutants

**Figure 2:**
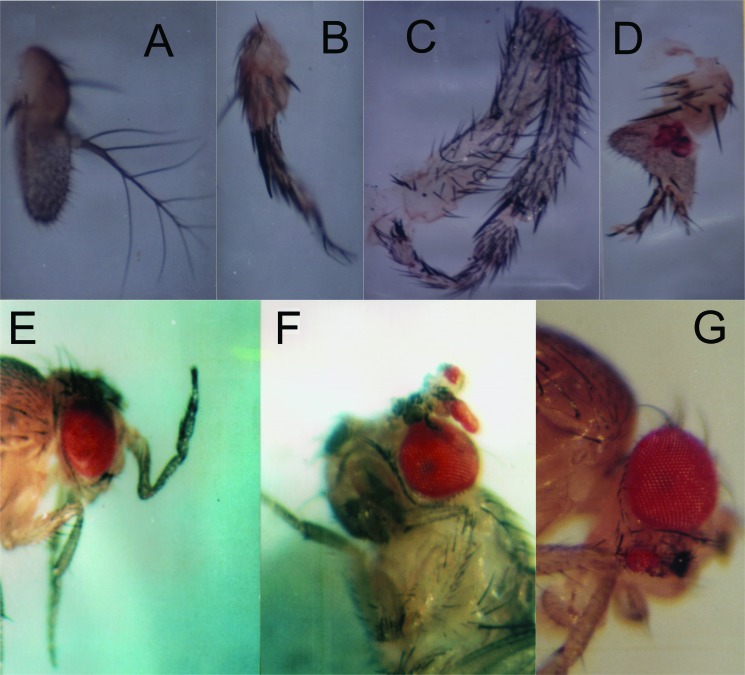
Variable expression of the *Zp* (*Zoinho-na-pata*) mutant. This mutant shows ectopic expression of legs in the antennae and, sometimes the expression of ectopic eyes. The mutation is dominant, but some individuals show normal antennae (A), a weak transformation of antennae to leg (B-C), a complete leg in place of antenna (E), or eye structures in the ectopic leg (D, F, G).

We have characterized a hypermutable strain of *Drosophila simulans* (Dshs), originated from a single spontaneous mutant male, collected in nature, showing the *lozenge* phenotype. The genetic characterization of this mutant revealed that the females are sterile due absence of spermathecae. Therefore, to maintain the mutants in the laboratory, the males were crossed with a wild strain (*D. simulans* Eldorado). During this process, new mutations were observed. The strain was followed for roughly 100 generations, and during the mutation screening, several of the isolated mutants corresponded to developmental genes (Loreto *et al.*, 1998). One interesting mutant, which can represent the potential of transposons to create “evolutionary novelties” is the one showing an antennapedia phenotype, where legs grow in place of antennae. In addition, in this particular mutant, ectopic eyes grow on homeotic legs. This allele is dominant, and flies show a phenotype with variable expressivity, ranging from normal antennae to homeotic legs, with approximately 6% of flies expressing ectopic eyes on the homeotic legs (Figure 2). This gene was mapped to the 3L chromosome in the region corresponding to the *eyegone* locus, although no molecular evidence has confirmed the mutation’s presence in this gene (unpublished result).

**Figure 3:**
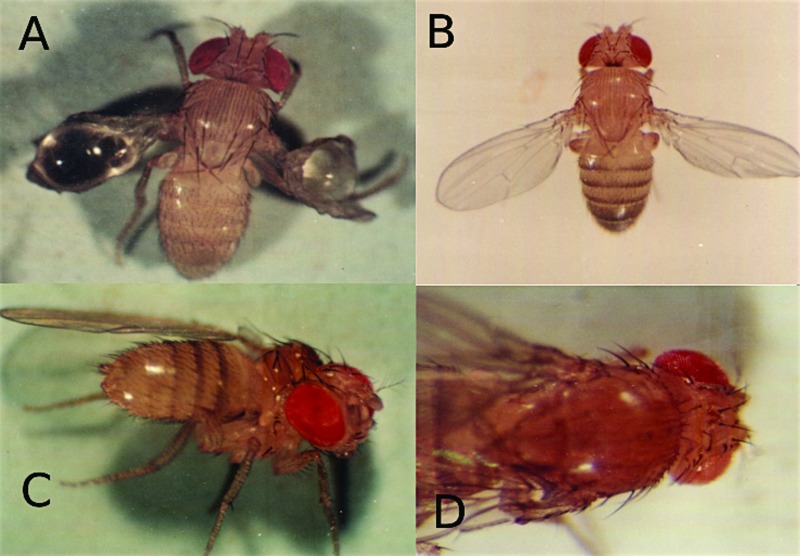
Mutant phenotypes A) Phenotypic appearance of *blistered* mutant (*bl*); B) *dpp* mutant; C) somatic mutation in which half the thorax and one wing was not formed; D) mosaic fly in which one eye has a wild type phenotype and the other has a *lozenge* appearance.

Other mutations were characterized and mapped to, for example, the *decapentaplegic* gene (*dpp*); *lozenge* (*lz*); *blistered* (*bs*); and *white* (*w*) (Figure 3) (Loreto *et al.*, 1998; Torres *et al.*, 2006). The *blistered (bl)* mutant (Figure 3A) is dominant, showing incomplete penetrance, which is sensitive to temperature, with stronger expression at higher temperatures. The same increase in phenotypic expression was observed in the *Zp* mutant (Loreto ELS, 1997, Doctoral thesis, Universidade Federal do Rio Grande do Sul, Porto Alegre, RS). The mutant *decapentaplegic (dpp)* is recessive, and the homozygous flies have wings, which are held out laterally (Figure 3B).

In the hypermutable strain, the occurrence of a high rate of somatic mutations is suggested, as it has been observed in many flies with severe phenotypic alterations that are not inherited (Figure 3C) (Loreto *et al.*, 1998).

## The putative causal agent of this hypermutability: an old, hectic, energetic and degenerated hobo element

**Figure 4:**
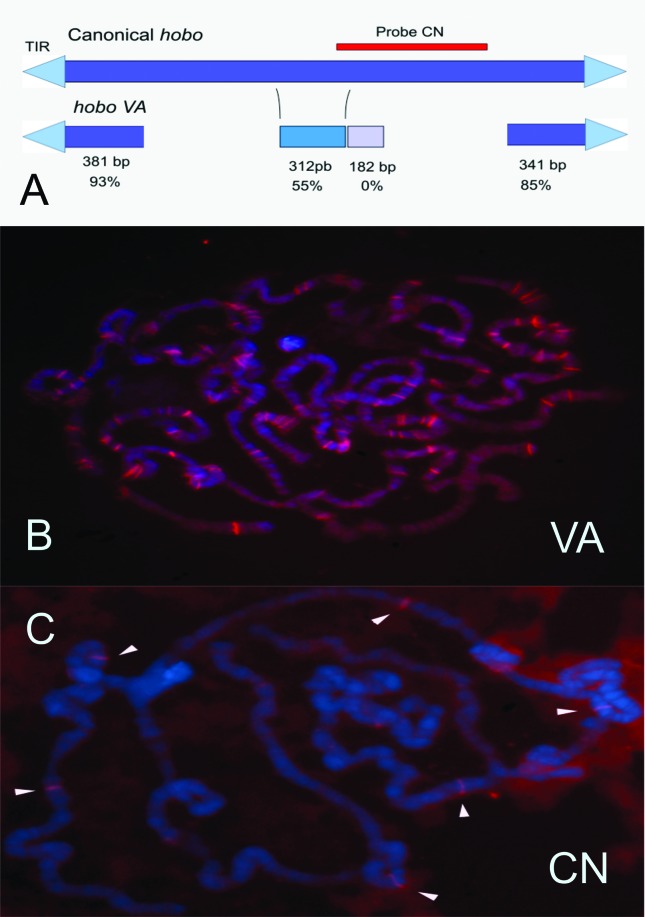
Canonical *hobo* and *hoboVA*. A) Schematic representation of canonical *hobo* and *hobo*VA. TIR are represented as a triangle. The red line shows the probe region used in *in situ* hybridization (C). The *hobo*VA element has the best conserved extremities and a more divergent inner sequence. The similarity for each region is indicated in %, and the sizes of the regions are indicated in base pairs (bp); B) *in situ* hybridization of polytene chromosomes using the complete *hobo*VA as probe; C) *in situ* hybridization of polytene chromosomes using the inner portion of canonical *hobo* as probe. Arrows point to the hybridization sites.

The molecular characterization of a “*de novo” white* mutation isolated in the hypermutable strain showed that it was caused by an insertion of a “relic” non-autonomous *hobo* element. This is a 1.2 kbp element, with conserved regions of 12 bp TIRs, 8 bp TSD, and subterminal sequences (Figure 4A). The 5’ region was 381 bp in length and showed 93% similarity with canonical *hobo.* The 3’ region was 341 bp in length and 85% similar to canonical *hobo.* The inner region is AT rich and has low similarity with canonical *hobo* (Torres *et al.*, 2006). This non-autonomous element is mobilizable in the hypermutable strain, and it is involved in “*de novo*” mutations and contains sufficient sequences for transposition (a minimum of 141 bp on the 5’ end and 65 bp on the 3’ end) (Kim *et al.*, 2011). The source of transposase to induce mobilization is postulated as the canonical *hobo*, which is present in this strain (Torres *et al.*, 2006; Deprá *et al.*, 2009).

Although the only mutation for which the causal agent was fully characterized as being the *hobo*VA element was the *white* mutant, two other facts lead us to suggest that the causal agent of the hypermutability in this strain is the *hobo*VA. First, an insertion of the same size of that element, 1.2 kbp, was also observed in the *lozenge* mutant generated in this hypermutable strain (Loreto *et al.*, 1998). Second, it has long been known that the *cis*-regulatory heldout region of the *decapentaplegic* (*dpp*) gene is a preferential site for *hobo* insertions (Newfeld and Takaesu, 1999). One of the mutants we have isolated is with the heldout phenotype of *dpp*.

Aiming to verify the abundance of sequence similar to *hobo*VA in the *D. simulans* genome, we performed an *in silico* analysis on the genome available after the publication of the 12 *Drosophila* genomes by Clark *et al.* (2007). In that study, the genome of *D. simulans* was assembled using a mix of seven strains. The analysis showed that these 1.2 kbp sequences, similar to *hobo*VA, are abundant, with 147 copies scattered across all chromosomes. These comprise 92 putatively mobilizable sequences and 72 with TSDs, indicative of recent mobilization. However, the sequenced strains only had two copies of the putative autonomous *hobo* element (Ortiz and Loreto, 2008). Also, we have performed a quantification of *hobo*VA sequences in our hypermutable *D. simulans* strain, showing that this element is also abundant in the strain. Figure 4B shows the fluorescent *in situ* hybridization (FISH) of polytene chromosomes with the *hobo*VA element, where at least 90 hybridization sites can be identified. In contrast, when the polytene chromosomes were hybridized with the inner portion of the *hobo* element, found exclusively in the complete elements, only six hybridization sites were observed (Figure 4C).

Another characteristic of these *hobo-*related elements, *hobo*VA, is that they have apparently been maintained for an evolutionary time that is prior to the *D. sechellia* and *D. simulans* speciation event, estimated at 0.4 MYA. Sequences similar to *hobo*VA are found in both species, suggesting that this element has been maintained as a non-autonomous element in the genomes of these species for all this time (Torres *et al.*, 2006; Ortiz and Loreto, 2008). The presence of short, non-autonomous but mobilizable elements in a higher number, contrasting with low copy numbers of autonomous elements, appears to be a pattern for *hAT* elements (Ortiz *et al.*, 2010).

The data described above suggest that the hypermutable strain could have an autonomous *hobo* element, free of silencing mechanisms, and in this way, able to mobilize *hobo*VA elements. Because these “relics” elements are maintained for a long time, and are very active, we call it *hobo* “Velho Assanhado” (VA), which in Portuguese means “a very animated elder”.

## hoboVA and his *cis*-regulatory developmental sites

**Figure 5:**
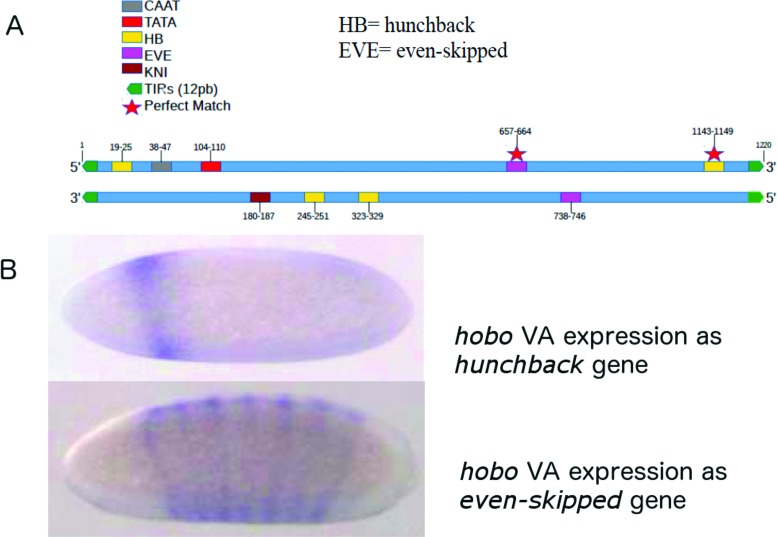
*hobo* transcriptional regulation. A) Transcription factor binding sites (TFBSs) in the *hobo*VA element predicted by “motility” toolkit. For this analysis, we searched for high scoring binding sites of six homeotic genes (*bicoid, even-skipped, fushi-tarazu, hunchback, knirps and kruppel*) using matrices described in Ho *et al.* (2009). Possible CAAT and TATA boxes were found using the description of *hobo* elements by Streck *et al.* (1986), for reference in the alignments. TFBSs are represented by colored boxes. A red star indicates a perfect match of TFBS and the *hobo*VA sequence; B) *in situ* hybridization whole-mount embryos of the *Drosophila simulans* hypermutable strain, using *hobo*VA as probe (RNA). *hobo*VA can be seen expressing as *hunchback* and *even-skipped* in two different developmental stages.

The transcription factor binding sites in the *hobo*VA element were predicted using the “motility” toolkit, which allowed us to search for sequence motifs using position weight matrices. For this analysis, we searched high scoring binding sites for six homeotic genes (*bicoid, even-skipped*, *fushi-tarazu, hunchback, knirps* and *krüppel*) using matrices described in Ho *et al.* (2009). Max-scoring matches were found for *even-skipped* and *hunchback* (Figure 5A).

Experimental evidence that the *cis*-regulatory sites for *hunchback* and *even-skipped* are functional in *hobo*VA were shown by Deprá *et al.* (2009). *In situ* hybridization in embryos of flies belonging to hypermutable and other strains, using *hobo*VA as a probe showed expression comparable to that observed for *hunchback* and *even-skipped* (Figure 5B).

The presence of *cis-*regulatory sequences of developmental genes, mainly those expressed in the initial phase of embryonic development, have been described for many TEs. For example, several retrotransposons of *Drosophila* have these sequences (Ding and Lipshitz, 1994; Borie *et al.*, 2002), as do LINEs in mammals (Loh *et al.*, 2006; Gerdes *et al.*, 2016). Transcription factor binding sites related to genes involved in the initial phases of development can be selectively advantageous for TEs, which could maximize their chance of increasing their presence in the next generation. For organisms whose germlines and somatic cells are separated, it is important for TEs to be active in phases when transposons can increase their copy number in the germ line, but not in somatic cells. Transposition in germ cells can be selectively advantageous from TE perspectives, yet transposition is normally detrimental in somatic cells (Haig, 2016).

## Creating new regulatory networks

From Figure 5B, it can be seen that *hobo*VA are scattered across all chromosomes, carrying its regulatory sequences. When some of these transposons are mobilized, they can be inserted in nearby genes, leading to a new position for their transcription factor binding sites, and this can modify the expression of genes in these new locations. Although we do not have a molecular characterization of the *Zoinho-na-pata (Zp)* mutation, we can hypothesize that the ectopic expression of *Zoinho-na-pata* mutants, as well as other mutants observed in the hypermutable strain, could be a product of *hobo*VA insertion. Master control genes, such as *eyegone,* which is involved in antennal and eye development and morphogenesis (Dominguez *et al.*, 2004; Yao and Sun, 2005; Wang *et al.*, 2008), can activate a new spatiotemporal pattern of gene expression when they receive insertions of new *cis*-regulatory sequences in their regulatory region. Therefore, transcription factor binding sites (TFBSs) for *hunchback* and *even-skipped,* present in *hobo*VA, can produce new phenotypes if inserted in such genes. These TFBSs are known as promoters of spatiotemporal gene control compatible with those observed in *Zp* mutant phenotype.

From an evolutionary point of view, the spread of *cis*-regulatory sequences can rewire gene regulatory networks. This can occur with the gradual addition of these sequences in the promotor regions of new genes, such as products of new TE insertions. As consequence of these insertions, genes can show new regulatory patterns by answering to transcription factors in which they were not respondent before. This rewiring can later undergo fine-tuning, resulting by natural selection of other mutations in the involved genes and the regulatory sequences that were added to the system by TEs. The involvement of TEs in rewiring gene networks is well supported in the literature (Feschotte, 2008; Feschotte and Gilbert, 2012; Rebollo *et al.*, 2012; Chuong *et al.*, 2017). The classical Darwinian view of evolution as a gradual process, in which no leaps are taken, fits well in this scenario of the rewiring of gene networks. Also, it has been shown in the literature that complex structures can evolve gradually as, for example, complex organs such as eyes found in vertebrates, insects or cephalopods have evolved from photoreceptor cells, in which many intermediary steps can be found throughout the animals phylogeny (reviewed in Gehring, 2002).

The idea of large mutations producing great leaps of adaptation, as originally proposed by Richard Goldschmidt, in his *hopeful monster* theory, was refuted for a long time. Now, some examples indicate these “monsters” could have a place in evolutionary theory, though not exactly as frequently credited to Goldschmidt’s original proposition, as mutations with dramatic alterations in phenotype, producing an organism perfectly adapted to the environment. However, Chouard (2010) has revised some examples where single-gene changes promoting large phenotype effect can confer large adaptive value. These examples are not in disagreement with the Darwinian theory, they only open space for mutations with large phenotypic consequences, which, when viable in natural situations, could be initial steps for evolutionary novelties.

Master control genes are at the top of networks to build structures, body parts, and metabolic routes. Many master control genes are themselves transcription factors. When transposons carrying transcription factor binding sites (TFBSs) insert into the regulatory region of a master control gene, they can, theoretically, imbricate phenotypic building cascades, leading to evolutionary novelties.

The appearance of antennae with eyes could constitute a large evolutionary leap. Unfortunately, after some year of maintenance in the laboratory, we lost the *Zp* strains, making it impossible to show if *hobo*VA was involved in this particular mutation. The difficulty in maintaining this strain in the laboratory is `per se’ indicative that such mutations normally are inviable in nature. However, we can imagine that insertions of TEs, such as *hobo*VA, carrying TFBSs for master control genes, can bring new regulatory patterns for other master control genes, producing new phenotype patterns. If so, maybe some “hopeful monsters” could be the products of TE insertions, as is suggested by this hypothetical example. Maybe, *hopeful monsters* need a “lucky spot”. Large phenotypic alteration, when occurring in particular environments, could be the initial point for evolutionary novelties, and TEs can be part of this process.
